# 2-Dodecyl-6-methoxycyclohexa-2,5-diene-1,4-dione inhibits the growth and metastasis of breast carcinoma in mice

**DOI:** 10.1038/s41598-017-07162-3

**Published:** 2017-07-27

**Authors:** Chunxia Chen, Zhihuan Nong, Qiuqiao Xie, Junhui He, Wene Cai, Xiuneng Tang, Xiaoyu Chen, Renbin Huang, Ying Gao

**Affiliations:** 1 Department of Pharmacology, Guangxi Medical University, Nanning, Guangxi 530021 China; 2 Department of Biology and Tennessee Center for Botanical Medicine Research, Middle Tennessee State University, Murfreesboro, TN 37132 USA

## Abstract

Metastasis causes approximately 90% of breast cancer-related deaths in women. Previously, we have demonstrated that 2-dodecyl-6-methoxycyclohexa-2,5-diene- 1,4-dione (DMDD) remarkably inhibited the growth of human breast cancer cells with little toxicity. In this study, we investigated the toxicity and efficacy of DMDD to treat metastatic breast tumors using an *in vivo* mouse model of the 4T1 mammary carcinoma. DMDD caused no observable toxicity and significantly extended the survival of 4T1 tumor-bearing mice. DMDD effectively inhibited the growth of 4T1 cells *in vitro*, and suppressed the growth and metastasis of mammary tumor *in vivo*. The levels of inflammatory cytokines in the serum (TNF-α, IL-6, IL-12, TGF-β, and VEGF) were down regulated by DMDD. Immunohistochemical analysis demonstrated that the inhibition of tumor growth and metastasis was associated with activation of Bax, cleaved caspases-3 and -9, and down-regulation of Bcl-2, MMP-2 and -9, NF-κB and IκBα. We speculate that DMDD inhibits cytokine production in the tumor cells in mice, which leads to deactivation of NF-κB pathway, and consequently inhibits the expression of many anti-apoptosis and metastasis-promoting genes, such as Bcl-2 and MMPs. Collectively, our results demonstrate the potential of DMDD as a safe and effective antitumor agent in the treatment of late-stage breast cancer.

## Introduction

Breast cancer is the most common type of cancer and the second leading cause of cancer deaths among women, with approximately 244,700 new cases and 39,400 estimated deaths in the United States in 2016^1^. Notably, the risk of a woman developing breast cancer over her lifetime is one in eight^1^. Despite the improvement in survival rates of women who are diagnosed with breast cancer due to early detection and advanced treatment, breast cancer remains a huge health threat to women worldwide. Particularly, metastatic breast cancer, or stage IV breast cancer, which is the spread of malignant tumors from breast to other organs (primarily lung, liver, bone, and brain) in the body, causes approximately 90% of breast cancer-related deaths in women^2^. Unfortunately, metastatic tumors are usually inaccessible by surgery or radiotherapy, and there are currently no effective therapies to treat breast cancer metastasis^3^. Therefore, it is essential to develop new therapeutic agents to prevent and treat metastatic breast cancer.

Accumulating evidence has shown that several inflammatory cytokines, such as TNF-α, interleukin-6 (IL-6), interleukin-12 (IL-12), transforming growth factor β (TGF-β), and vascular endothelial growth factor (VEGF), are involved in the initiation and progression of cancer^4–8^. These inflammatory cytokines are released by immune cells in response to triggering factors, such as chronic inflammation, and mediate a sequential cascade of cellular events, including the generation of reactive oxygen species (ROS) and reactive nitrogen species (RNS)^9, 10^, sustained activation of tumor-associated signaling pathways such as nuclear factor κB (NF-κB) pathway^11^, epithelial-mesenchymal transition (EMT)^12–14^, and inflammation-associated angiogenesis^15^. These cellular events eventually lead to the elimination of antitumor immunity and acceleration of tumor progression^4^.

The metastasis of tumors is a complex process that involves multiple steps, which are termed the “invasion-metastasis cascade”, including initial separation from the primary tumor, local invasion through the stromal extracellular matrix (ECM), intravasation into blood vessels, survival during circulation, arrest and extravasation at distant tissues, adaptation to the foreign microenvironment and re-initiation of the proliferation^16, 17^. Matrix metalloproteinases (MMPs) are the key molecules in the degradation of the stromal ECM, which is one of the most crucial steps for tumor metastasis^18^. Among the six subgroups of the MMP family (collagenases, gelatinases, stromelysins, matrilysin, elastase, and membrane-type MMPs), MMP-2 and -9 are two gelatinases that degrade denatured collagen and native type I, IV, V collagen^19, 20^. The expression of MMPs, including MMP-2 and -9, is primarily regulated by activator protein-1 (AP-1) and NF-κB^21, 22^.

NF-κB signaling is required in both tumor cell proliferation and metastasis^23, 24^. NF-κB is a transcription factor that is retained in the cytoplasm by inhibitor κB α (IκBα). Upon induction by TNF-α, IκBα is phosphorylated and degraded, resulting in the dissociation of NF-κB p65 from IκB and the translocation of NF-κB p65 into the nucleus^21, 22^. Consequently, activated NF-κB p65 promotes the transcription of genes that regulate cancer cell proliferation, angiogenesis, and metastasis, such as the Bcl-2 family anti-apoptosis genes and the MMP family genes^21, 25^. Therefore, targeting the NF-κB signaling pathway has been considered a promising strategy to prevent tumor growth and metastasis^26^.

*Averrhoa carambola L*. (Oxalidaceae), commonly referred to as the starfruit tree, is native to and widely distributed in Southeast Asia. The root of *Averrhoa carambola L*. has been used in traditional Chinese medicine (TCM) to treat arthralgia and chronic paroxysmal headaches for thousands of years^27^. Previously, we isolated a new compound, 2-Dodecyl-6-methoxycyclohexa-2,5-diene-1,4-dione (DMDD) from *Averrhoa carambola L*. root extract, and showed that DMDD exerted anti-diabetic effects *in vivo* ^28^. Recently, we demonstrated *in vitro* that DMDD had anti-proliferation efficacy against a variety of human cancer cells lines, including A549, BT20, MCF-7, MDA-MB-231 and U2OS^29^. Utilizing human breast cancer cell lines MCF-7 and BT20, we showed that DMDD exerted its inhibitory activity through cell cycle arrest, induction of apoptosis and oxidative stress. We also showed that the *in vitro* inhibitory effect of DMDD on NF-κB activation occurred due to the prevention of the phosphorylation of IκBα and the nuclear translocation of NF-κB, which consequentially down-regulated the anti-apoptotic molecules and promoted apoptosis in human breast cancer cells^29^. However, the *in vivo* anti-tumor and anti-metastasis effects of DMDD against breast cancer have not yet been investigated.

It is notable that DMDD showed no or little toxicity to mice and normal human cells. Administration of DMDD at up to 50 mg/kg body weight for consecutive eight weeks did not show a significant difference in the renal function compared with the vehicle^28^. DMDD also exhibited significant lower toxicity to the normal human cells versus corresponding human cancer cells^29^. Because DMDD exhibited superior anti-tumor efficacy *in vitro* without observable toxicity, in the present study we have investigated the *in vivo* anti-tumor and anti-metastasis efficacy of DMDD using a 4T1 metastasis breast cancer model. We determined the effects of DMDD on the production of inflammatory cytokines and the TNF-α/NF-κB/MMPs pathways. This study expands our knowledge of the therapeutic potentials of DMDD for the treatment of breast cancer, especially metastasis breast cancer.

## Results and Discussion

### DMDD Caused No Observable Toxicity to Mice

During the course of the acute toxicity test, there were no apparent abnormalities observed, such as vomiting, diarrhea, hair removal and death in both groups. The body weights of mice in the DMDD group were steadily increased. There was no significant statistical difference in the body weight and food consumption between the DMDD group and the control group (*p* > 0.05) (Table 1).

**Table 1:** Effect of single dose of 5,000 mg/kg DMDD on the body weight and food consumption in mice (n = 10).

	Body weight (g)			Food consumption (g/mouse/day)		
	0 week	1 week	2 weeks	0 week	1 week	2 weeks
Control group	19.68 ± 1.01	22.40 ± 1.25	25.46 ± 1.17	4.20 ± 0.19	4.94 ± 0.20	5.62 ± 0.24
DMDD group	20.06 ± 0.97	23.13 ± 1.11	25.84 ± 1.13	4.11 ± 0.29	4.88 ± 0.28	5.60 ± 0.29

Next, the hematological and biochemical parameters of the blood samples were examined. There was no significant statistical difference in all hematological parameters between the DMDD group and the control group (*p* > 0.05), except HB (*p* < 0.05) (Table 2). However, the amount of HB in the DMDD group (160.95 ± 7.99 g/L) is within the normal range for healthy mice^30^. There was no significant statistical difference in all blood biochemical parameters between the DMDD group and the control group (*p* > 0.05) (Table 3).

**Table 2:** Effect of single dose of 5,000 mg/kg DMDD on blood hematological profile in mice (n = 10). *p < 0.05.

Parameters	Control group	DMDD group	*p* Value
RBC (10^12^/L)	10.03 ± 1.70	9.44 ± 2.02	0.49
HB (g/L)	154.25 ± 4.73	160.95 ± 7.99	0.03*
MCH (pg)	15.13 ± 0.54	15.13 ± 0.94	1.00
MCHC (g/L)	326.92 ± 12.34	320.85 ± 7.09	0.19
MCV (fL)	49.36 ± 3.43	50.19 ± 3.53	0.60
PLT (10^9^/L)	461.30 ± 46.73	503.02 ± 144.89	0.40
WBC (10^9^/L)	5.71 ± 1.17	4.54 ± 1.95	0.37
NEUTR (%)	2.74 ± 0.43	3.08 ± 1.15	0.40
LYR (%)	80.77 ± 13.57	87.62 ± 9.54	0.21

**Table 3:** Effect of single dose of 5,000 mg/kg DMDD on blood biochemical parameters in mice (n = 10).

Parameters	Control group	DMDD group	*p* Value
ALT (U/L)	57.09 ± 17.18	69.54 ± 13.33	0.09
AST (U/L)	187.01 ± 56.35	208.66 ± 39.40	0.33
ALP (g/L)	170.90 ± 29.58	186.70 ± 33.80	0.28
TP (g/L)	68.72 ± 4.21	71.34 ± 4.18	0.18
ALB (g/L)	30.83 ± 4.93	33.38 ± 3.97	0.22
CREA (μmol/L)	16.71 ± 4.98	14.40 ± 1.73	0.18
BUN (mmol/L)	8.61 ± 3.37	7.99 ± 1.48	0.60
TCh (mmol/L)	2.29 ± 0.58	2.38 ± 0.51	0.71
TG (g/L)	2.15 ± 0.78	2.06 ± 0.74	0.80
GLU (mmol/L)	5.67 ± 0.58	6.14 ± 1.03	0.23

Additionally, there was no noticeable histopathological change observed in the vital organs such as heart, liver, lung and kidney of the mice in the DMDD group compared with these of the control group (Supplementary Figure 1). Overall, the data suggested that administration of a single dose of 5,000 mg/kg DMDD did not show toxicity to mice.

### DMDD Treatment Extended the Survival of 4T1 Xenograft Mice

**Figure 1:**
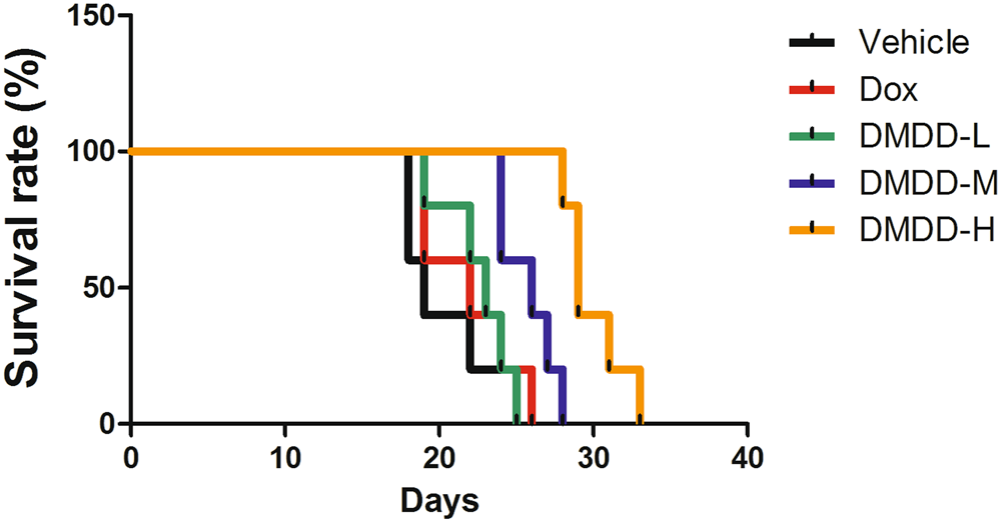
Effect of DMDD on the survival of 4T1-induced breast cancer mice. Female BALB/c mice were injected with 10^6^ 4T1 cells on their right mammary fat pads. DMDD was administrated to the mice for 14 days. The survival time (days) for each mouse was recorded.

The survival curve for each group was shown in Fig. 1. The survival time of the vehicle group, DOX group, DMDD-L group, DMDD-M group and DMDD-H group was 20.5 days, 22.5 days, 23.3 days, 26.5 days, and 29.5 days (*p* = 0.002), respectively. DMDD in low, medium and high dose extended the survival by 13.7%, 29.3%, and 43.9%, respectively. The results showed that DMDD significantly extended the overall survival of 4T1 tumor-bearing mice.

### DMDD Treatment Inhibits the Growth of Primary Mammary Tumors in 4T1 Xenograft Mice

**Figure 2:**
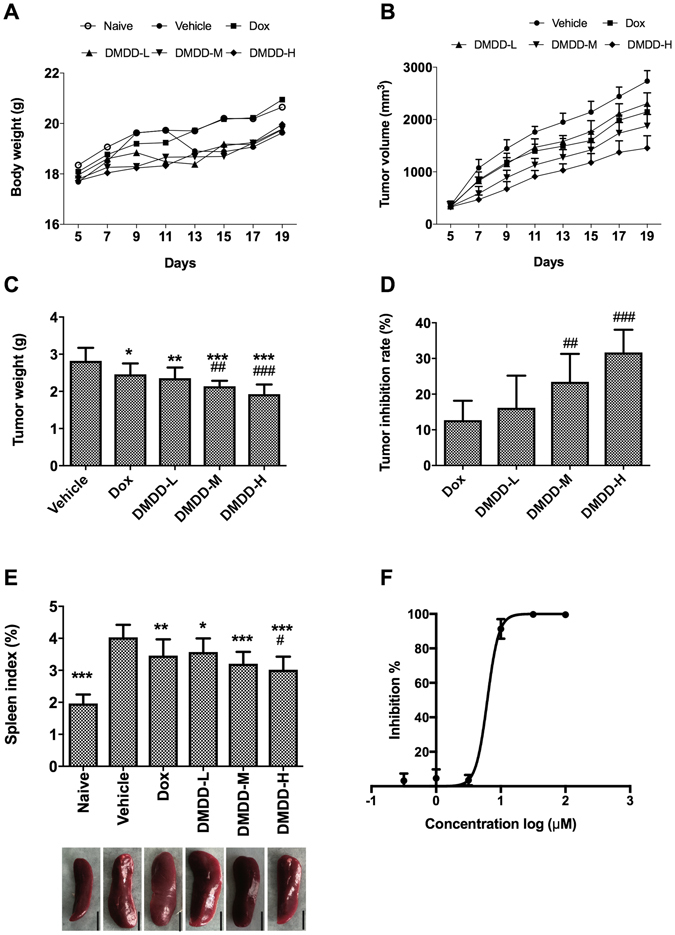
*In vivo* and *in vitro* inhibitory effects of DMDD on the growth of 4T1 mammary tumors. Female BALB/c mice were injected with 10^6^ 4T1 cells on their right mammary fat pads. When tumor volumes reached about 300 mm^3^, DMDD was administrated to the mice for 14 days. After that, the mice were euthanized, and tumor and spleen were isolated. (**A**) Body weights of the mice in each group over the experimental period. (**B**) Tumor volumes of the mice in each group as a function of time. (**C**) Tumor weights of the mice in each group at the end of the experiment. (**D**) Tumor inhibition rates of the various treatments. (**E**) Spleen index of each group at the end of the experiment. Data is presented as means ± standard deviation (n = 10). *p < 0.05 vs. vehicle group, **p < 0.01 vs. vehicle group, ***p < 0.001 vs. model group; ^#^ *p* < 0.05 vs. Dox group, ^##^ *p* < 0.01 vs. Dox group, ^###^ *p* < 0.001 vs. Dox group. (**F**) *In vitro* cytotoxic effect of DMDD in 4T1 murine mammary carcinoma cells. Cells were treated with various concentrations of DMDD for 48 h, and cell viability was accessed using the AlarmaBlue assay.

The 4T1 mouse mammary carcinoma cell line is a triple-negative breast cancer (TNBC) cell line and is documented to be very aggressive and highly metastatic^31^. Primary mammary tumors established in BALB/c mice using the 4T1 cell line is a well-recognized model to mimic metastatic breast cancer or stage IV breast cancer, which typically metastasizes to the lung and liver^3, 32^. In the present study, we used *in vivo* 4T1 metastatic mammary cancer models to determine whether administration of DMDD could inhibit primary tumor growth and metastasis in the lung and liver. The systemic toxicity and antitumor efficacy of DMDD were assessed in a 4T1 mammary tumor model by measuring the body weights and the tumor volumes of mice, respectively. As shown in Fig. 2A, all groups of mice were alive and showed no loss of body weight throughout the treatment period, including mice treated with Dox and DMDD at different dosages, even at a high dosage of 100 mg/kg. Body weight was an indicator to monitor the adverse effects of various treatments. These results further confirmed that there was no noticeable systemic toxicity caused by DMDD in addition to the acute toxicity test.

Tumor volumes were measured throughout the experiment. As shown in Fig. 2B, all treatment groups caused a significant reduction in the tumor volumes in a dose-dependent manner. Compared with the vehicle group that was treated with PBS only (2,441 ± 179 mm^3^), DMDD at doses of 25, 50, and 100 mg/kg decreased the tumor volumes to 2,114 ± 189 mm^3^ (*p* < 0.001), 1,739 ± 204 mm^3^ (*p* < 0.001) and 1,373 ± 221 mm^3^ (*p* < 0.001), respectively. Noticeably, DMDD at a dose of 25 mg/kg showed similar antitumor efficacy as the positive control Dox (1,988 ± 101 mm^3^, *p* < 0.001).

Tumor weights were measured, and the tumor inhibition rates were calculated at the termination of the experiment. As shown in Fig. 2C,D, all treatment groups caused significant inhibition of the tumor weights in a dose-dependent manner compared to the vehicle group. Compared with the vehicle group that was treated with PBS (2.822 ± 0.350 g), DMDD at doses of 25, 50 and 100 mg/kg decreased the tumor weights to 2.353 ± 0.287 g (*p* < 0.001), 2.140 ± 0.145 g (*p* < 0.001) and 1.923 ± 0.262 g (*p* < 0.001), respectively. The tumor inhibition rates of DMDD at doses of 25, 50 and 100 mg/kg were 16.2%, 23.5%, and 31.7%, respectively. Noticeably, DMDD at all doses showed higher antitumor efficacy than the positive control Dox (2.458 ± 0.294 g, 12.7%, *p* < 0.001). Particularly, DMDD at 50 and 100 mg/kg showed significantly higher efficacy than Dox (*p* < 0.01 and *p* < 0.001, respectively).

Overall, the administration of DMDD markedly reduced both the size and weight of tumors compared with the control group. These results suggest that DMDD has the ability to prevent the development of primary breast tumor in mice, with a higher efficacy than Dox.

Spleen index, which is the ratio of spleen weight against body weight, was calculated for each group. In 4T1 tumor-bearing mice, the spleens appeared enlarged, and the spleen index was significantly increased (*p* < 0.001). However, DMDD administration was able to reduce the elevated spleen index (*p* < 0.01 or *p* < 0.001), suggesting the protective effect of DMDD to the spleen, the primary immune organ of mice (Fig. 2E).

### DMDD inhibits cell proliferation in 4T1 mammary carcinoma cells

We tested the cell viability of 4T1 cells treated by DMDD. Treatment of 4T1 cells with DMDD at concentrations of 1-100 μM for 48 h resulted in a significant decrease in the cell viability. Particularly, less than 20% of 4T1 cells were survived after treatment with DMDD at concentrations of 10 μM and above. The inhibition curve is shown in Fig. 2F and the IC_50_ was determined to be 6.20 μM. Thus, the inhibitory effects of DMDD against 4T1 breast cancer cells are consistent both *in vitro* and *in vivo*.

### DMDD Treatment Decreases the Levels of TNF-α, IL-6, IL-12, TGF-β and VEGF in the Serum of 4T1 Xenograft Mice

**Figure 3:**
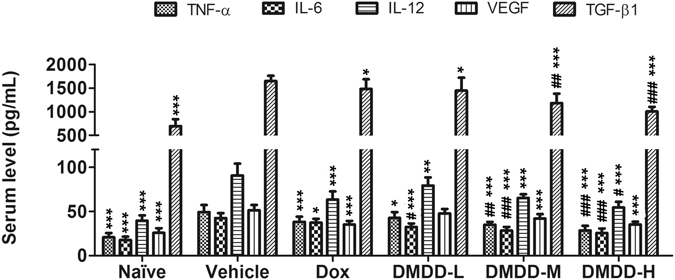
Serum cytokine levels measured with ELISA kits. Data is presented as means ± standard deviation (n = 10). *p < 0.05 vs. vehicle group, **p < 0.01 vs. vehicle group, ***p < 0.001 vs. model group; ^#^ *p* < 0.05 vs. Dox group, ^##^ *p* < 0.01 vs. Dox group, ^###^ *p* < 0.001 vs. Dox group.

Elevated levels of pro-inflammatory cytokines are closely associated with the initiation and progression of tumorigenesis^33, 34^. For example, TNF-α can activate IκBα phosphorylation and degradation, resulting in the dissociation and activation of NF-κB pathway. To determine whether DMDD affected cytokine production in the serum of 4T1 mice, we used ELISA kits to assess the level of TNF-α, IL-6, IL-12, TGF-β and VEGF after treatment with DMDD. As shown in Fig. 3A, DMDD caused a significant reduction in the levels of cytokines in the serum of mice (*p* < 0.05 or *p* < 0.01). All cytokines were suppressed by DMDD in a dose-dependent manner, and administration of DMDD at 100 μg/mL significantly reduced the production of all the five cytokines that were tested, TNF-α, IL-6, IL-12, TGF-β and VEGF, by 42.5%, 39.8%, 39.8%, 39.0% and 31.6% compared with the negative control, respectively.

### DMDD Treatment Alters the Histological Features of 4T1 Mammary Tumor tissues

**Figure 4:**
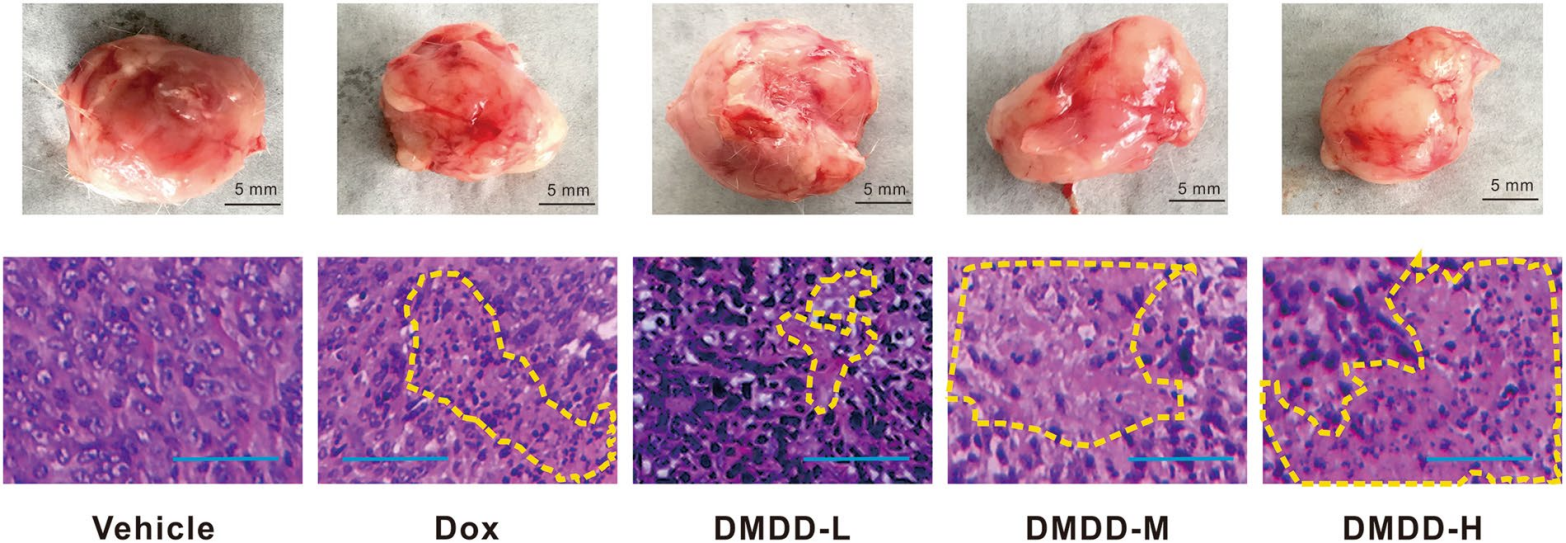
Histological analysis of 4T1 tumor sections. Tumor sections were stained with H&E. Upper panel: representative images of the mammary tumors in various groups at the end of the experiment. The scale bar represents 5 mm. Lower panel: representative H&E staining of the tumor sections. The nuclei were stained blue, and the cytoplasm and extracellular matrix were stained pink using H&E. The yellow dotted line shows the foci of tumor necrosis. The scale bar represents 100 μm.

To further investigate the antitumor efficacy of DMDD, the mammary tumors were dissected and sectioned for histological analysis at the end of the experiment. As shown in Fig. 4, in the tumor sections treated with vehicle, the tumor cells were packed tightly, distributed diffusely and interspersed with plentiful stroma. The tumor cells showed large nuclei and a clear nucleolus. However, after treatment with DMDD, the tumors exhibited significantly different histological properties. A decrease in the percentage of the nuclei indicated by blue spots was observed in all DMDD groups (DMDD-L, DMDD-M and DMDD-H) compared with the vehicle group. Meanwhile, nuclear shrinkage and fragmentation were also observed, indicating typical apoptotic characteristics induced by DMDD. Moreover, in the DMDD-M and DMDD-H groups, tissue necrosis and focal necrosis was observed. In the DMDD-H group, the necrosis area increased significantly, and only a small portion of tumor cells remained. These observations suggested a dose-dependent antitumor efficacy by DMDD.

### DMDD Treatment Induces Apoptosis of Tumor Cells in 4T1 Mammary Tumors

**Figure 5:**
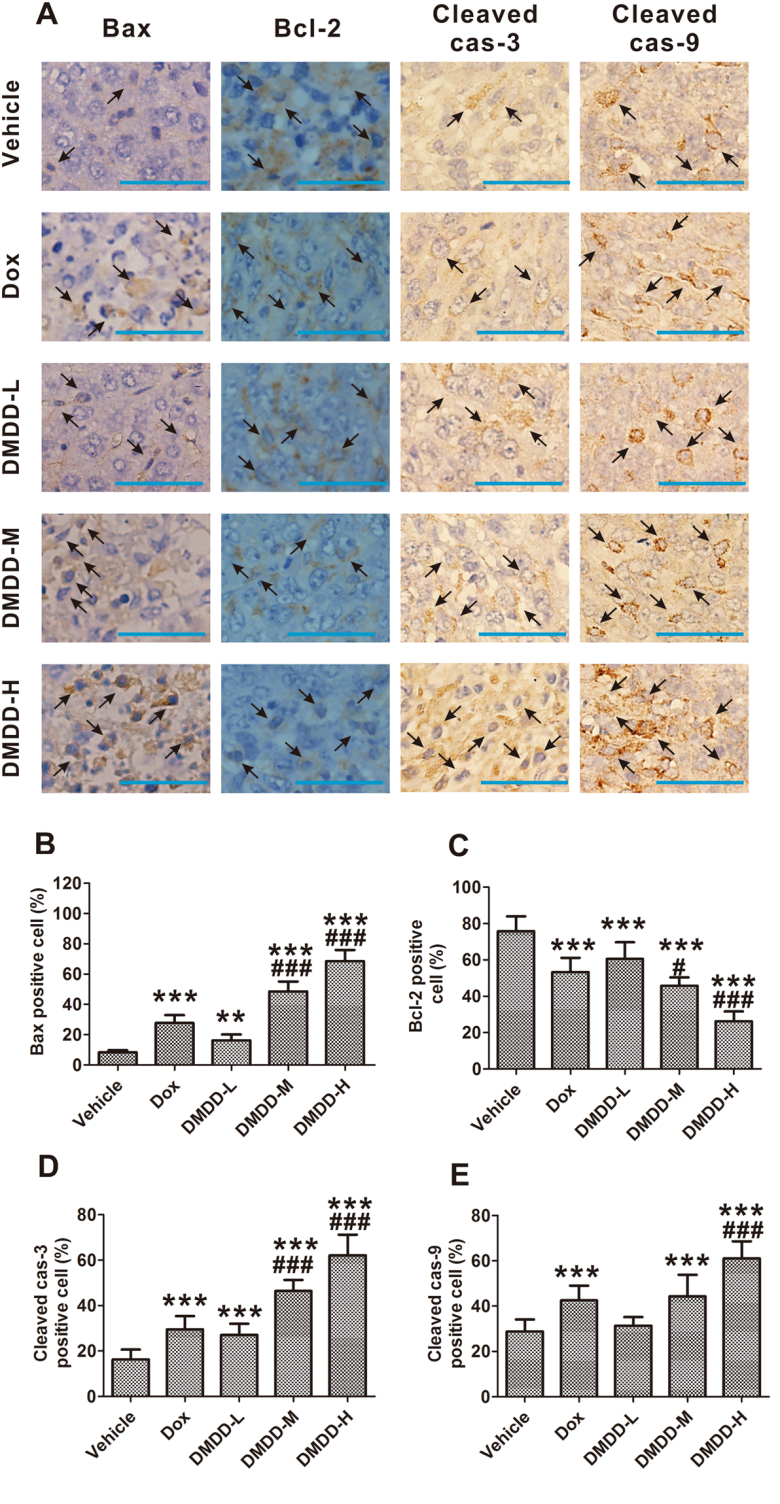
Apoptosis induction in the 4T1 tumor sections. Tumor sections were stained with DAB and antibody against Bax, Bcl-2, cleaved caspase-3 or cleaved caspase-9. (**A**) Immunohistochemical staining of 4T1 tumor sections. The nuclei were stained blue, and the target proteins were stained brown. The arrows point to the positive-staining cells. The scale bar represents 100 μm. (**B, C, D, E**) Quantification of the staining intensity. Data is presented as means ± standard deviation (n = 10). *p < 0.05 vs. vehicle group, **p < 0.01 vs. vehicle group, ***p < 0.001 vs. model group; ^#^ *p* < 0.05 vs. Dox group, ^##^ *p* < 0.01 vs. Dox group, ^###^ *p* < 0.001 vs. Dox group.

Apoptosis plays a crucial role in tumor development and progression by eliminating the cancerous and preneoplastic cells from the body. Histological analysis of the primary tumor tissue demonstrated that DMDD induced typical apoptotic characteristics, such as nuclear shrinkage and fragmentation^35^. We also conducted an immunohistochemistry analysis with the tumor sections to confirm the ability of DMDD to induce apoptosis *in vivo*. As apoptosis is modulated by a group of anti-apoptotic and pro-apoptotic regulators, we examined the expression of these key regulatory proteins in apoptosis in the tumor sections. Compared with vehicle control, administration of DMDD at 25-100 mg/kg significantly increased the number of cells that expressed cleaved caspase-3 and -9, which are characteristic hallmarks of apoptosis^36^ (Fig. 5A–C). B-cell lymphoma 2 protein (Bcl-2) and Bcl2 associated X protein (Bax) is an anti-apoptotic and pro-apoptotic regulator, respectively^37^. Administration of DMDD also significantly increased the number of cells that expressed Bax and decreased the number of cells that expressed Bcl-2 (*p* < 0.05) (Fig. 5A,D & E). These data showed that increased numbers of cells underwent apoptosis after DMDD treatment, suggesting that DMDD inhibited the breast tumor growth by induction of apoptosis.

### DMDD Treatment Inhibits the Expression of MMP-2 and -9 in 4T1 Mammary Tumors

**Figure 6:**
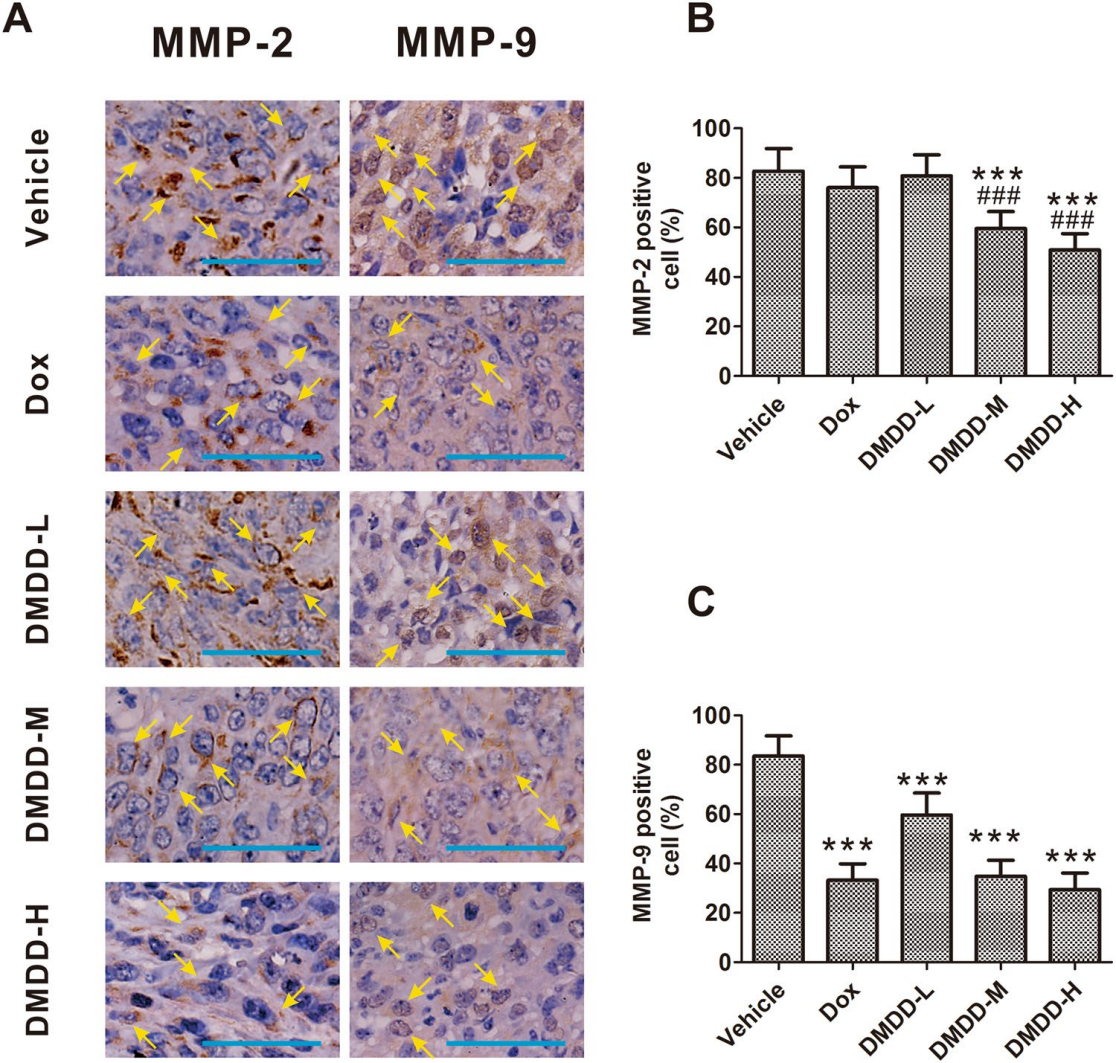
Expression of MMP-2 and -9 in the 4T1 tumor sections. Tumor sections were stained with DAB and antibody against MMP-2 or MMP-9. (**A**) Immunohistochemical staining of 4T1 tumor sections. The nuclei were stained blue, and the target proteins were stained brown. The arrows point to the positive-staining cells. The scale bar represents 100 μm. (**B, C**) Quantification of the staining intensity. Data is presented as means ± standard deviation (n = 10). *p < 0.05 vs. vehicle group, **p < 0.01 vs. vehicle group, ***p < 0.001 vs. model group; ^#^ *p* < 0.05 vs. Dox group, ^##^ *p* < 0.01 vs. Dox group, ^###^ *p* < 0.001 vs. Dox group.

MMP-2 and -9 are two key regulators that control tumor cell migration and invasion. Immunohistochemistry staining showed numerous brown-stained cells in the tumor sections treated with vehicle, which indicated that MMP-2 and -9 were both over expressed (Fig. 6A). However, after DMDD administration, both the numbers of MMP-2 positive cells and MMP-9 positive cells were decreased in a dose-dependent manner (Fig. 6B). The low-dose DMDD slightly decreased MMP-2 expression, while medium and high doses of DMDD decreased MMP-2 expression significantly compared with the vehicle control (*p* < 0.05), and showed even more potent effects than Dox (*p* < 0.05). Meanwhile, DMDD at all doses significantly decreased the expression of MMP-9 (*p* < 0.05).

### DMDD Treatment Suppresses the NF-κB Pathway in 4T1 Mammary Tumors

**Figure 7:**
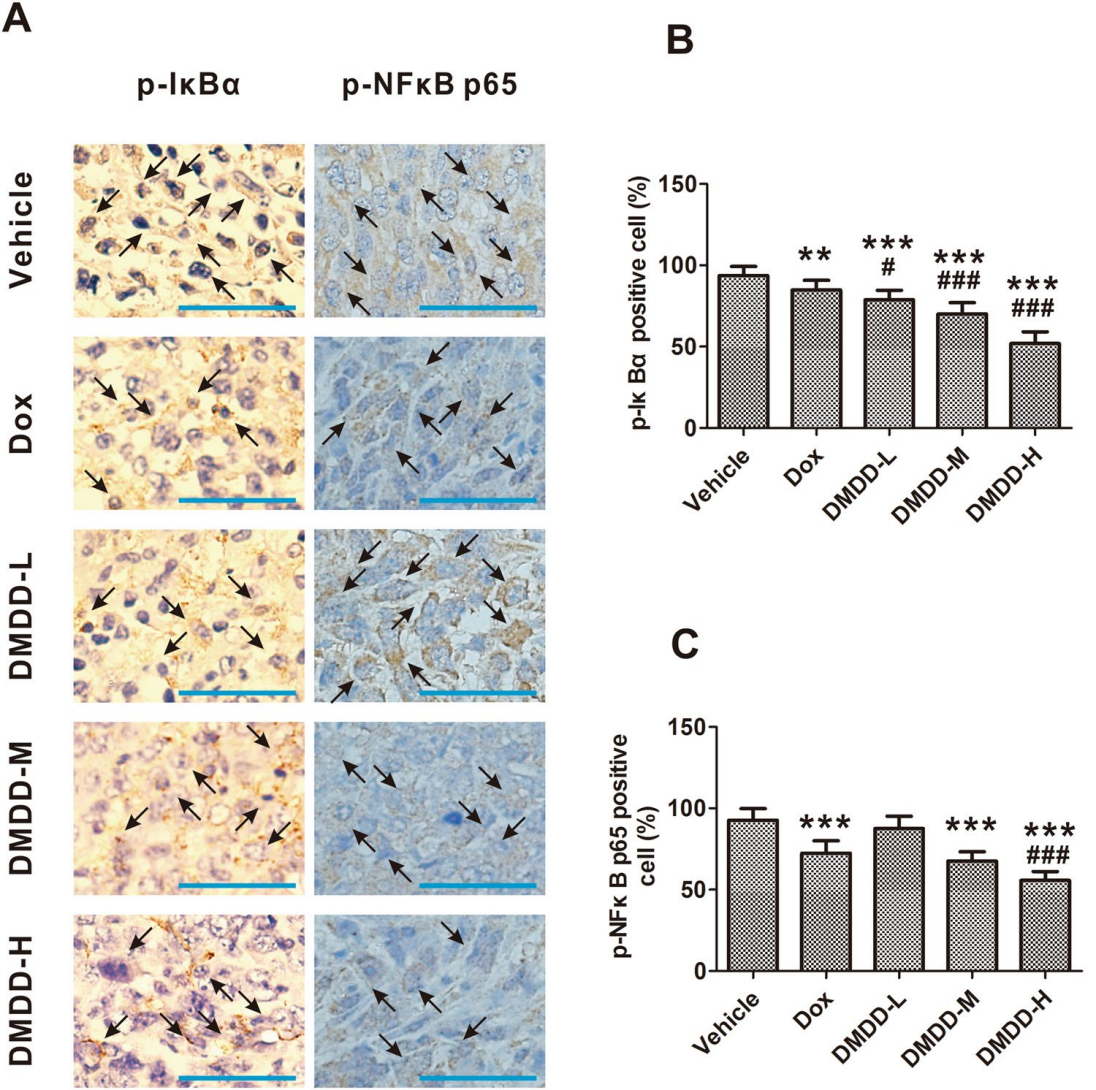
Expression of p-IκBα and p-NFκB p65 in the 4T1 tumor sections. Tumor sections were stained with DAB and antibody against p-IκBα or p-NFκB p65. (**A**) Immunohistochemical staining of 4T1 tumor sections. The nuclei were stained blue, and the target proteins were stained brown. The arrows point to the positive-staining cells. The scale bar represents 100 μm. (**B, C)** Quantification of the staining intensity. Data is presented as means ± standard deviation (n = 10). *p < 0.05 vs. vehicle group, **p < 0.01 vs. vehicle group, ***p < 0.001 vs. model group; ^#^ *p* < 0.05 vs. Dox group, ^##^ *p* < 0.01 vs. Dox group, ^###^ *p* < 0.001 vs. Dox group.

Immunohistochemistry staining of the primary tumor tissue also showed that DMDD administration significantly decreased the number of cells that expressed phosphorylated IκBα and phosphorylated NF-κB p65 in a dose-dependent manner (*p* < 0.05) (Fig. 7). DMDD at all doses showed significant inhibition on the phosphorylation of IκBα (*p* < 0.05), while DMDD at medium and high doses showed significant inhibition on the phosphorylation of NF-κB (*p* < 0.05).

As the phosphorylation of both NF-κB p65 and IκBα were down regulated after DMDD treatment, it indicated the inhibitory effect of DMDD on the NF-κB pathway. We speculate that DMDD treatment inhibits cytokine production in the tumor cells in mice, and leads to deactivation of NF-κB pathway, and consequently inhibits the expression of many anti-apoptosis and metastasis-promoting genes, such as Bcl-2 and MMPs. These *in vivo* observations were in agreement with our previous report on the *in vitro* inhibitory effect of DMDD on the NF-κB pathway in MCF-7 breast cancer cells^29^.

### DMDD Treatment Suppresses Lung and Liver Metastasis in 4T1 Xenograft Mice

**Figure 8:**
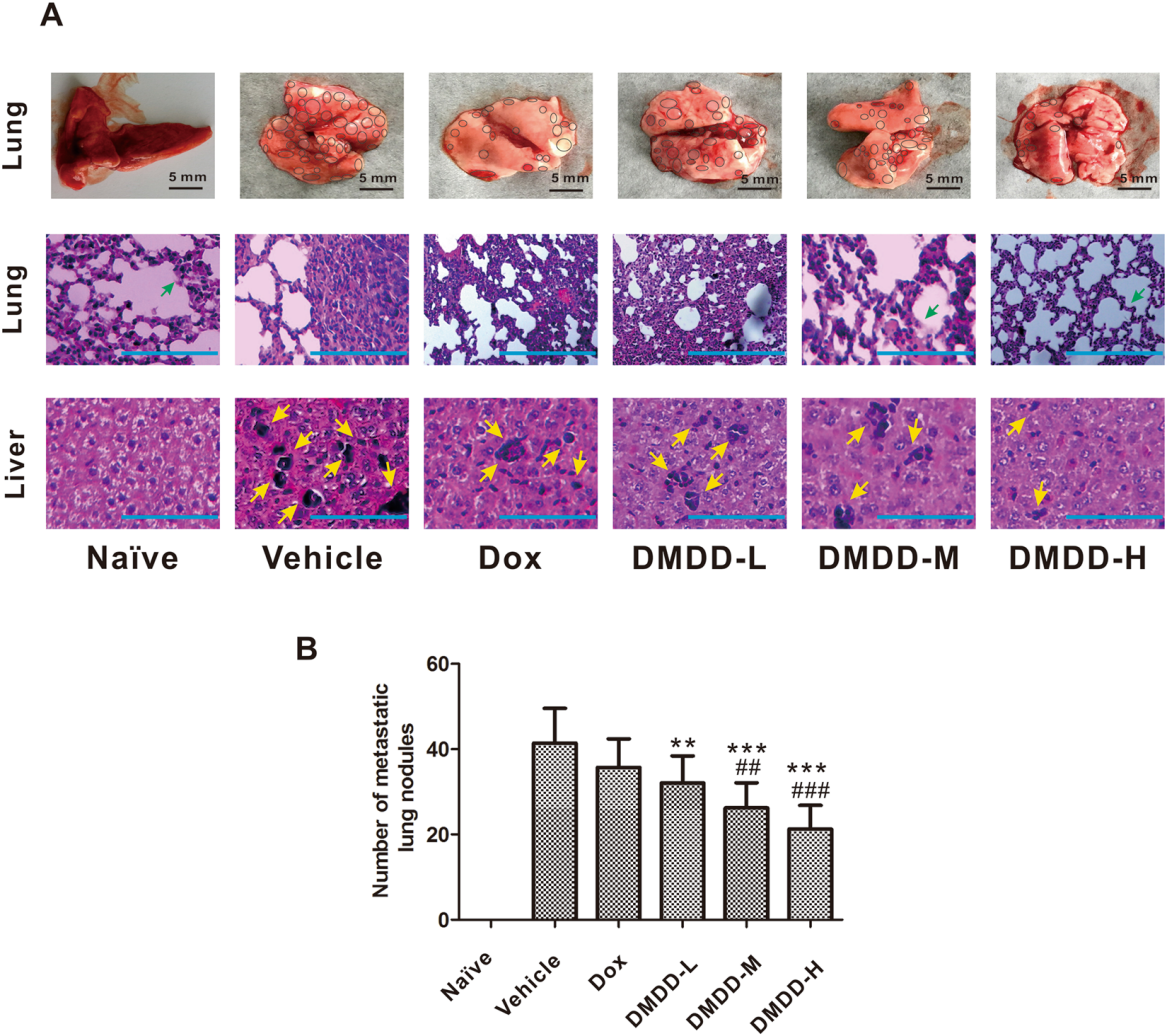
Histologic assessments of lung and liver with H&E staining in mice. (**A**) Top panel: representative images of the lung tissue in various groups at the end of the experiment. The scale bar represents 5 mm. The tumor nodules on the lung tissue are circled in black. Middle and bottom panels: representative H&E staining of the lung and liver sections. The scale bar represents 100 μm. The nuclei were stained blue, and the cytoplasm and extracellular matrix were stained pink using H&E. The yellow arrows point to the foci of tumor metastases, and the green arrows point to normal lung tissue. (**B**) Number of lung metastatic modules in mice.

The effects of DMDD on the lung and liver metastasis in 4T1 xenograft mice were also investigated. Lungs and livers from tumor-bearing mice were harvested and stained by H&E for immunohistochemistry analysis. In the vehicle group at the time mice were sacrificed, the primary tumor spread rapidly and massively to the lung and liver of the mice. A large number of tumor nodules were found in the lung tissue of mice, with the majority of the lung tissues occupied by tumor cells (Fig. 8A). However, decreased numbers of tumor nodules, as well as decreased size of tumor nodules, were observed in mice treated with DMDD at all dosages. Especially in the high-dose group, only a minimal level of metastasis in the lung was observed, and the tissues were left largely intact. DMDD at low, medium and high doses were sufficient to significantly decrease the numbers of tumor nodules compared with the vehicle control by 22.5%, 26.6% and 33.6%, respectively (*p* < 0.05), and the sizes of tumor nodules were obviously smaller in the treatment groups (Fig. 8B). The reduction in lung and liver metastases was confirmed by pathological examination, and the representative sections of lung and liver were also shown in Fig. 8A. Abundant metastatic foci occurred in the lungs and livers of mice treated with vehicle only, but less metastatic foci per field were observed in those treated with DMDD. In cancer patients, such pulmonary metastasis usually results in poor prognosis and quick death^38^. These data clearly showed that DMDD effectively inhibited lung and liver tumor metastases in addition to suppressing primary tumor growth in 4T1 tumor-bearing mice.

## Conclusion

In this study, significant anti-tumor and anti-metastasis effects of DMDD were observed in mice. First, we demonstrated that DMDD at both a single dose of 50,000 mg/kg and a consecutive dose of 100 mg/kg did not cause noticeable systemic toxicity in mice. Second, we showed that DMDD (25–100 mg/kg) extended the survival time of 4T1 tumor-bearing mice by up to 43.9% compared with the control. Third, we demonstrated that DMDD (25–100 mg/kg) significantly decreased the tumor volume by up to 43.8% and decreased tumor weight by up to 31.7% compared with the vehicle group. Lastly, we showed that DMDD also effectively suppressed the metastasis of the primary breast tumors to the lung and liver. In conclusion, the experimental results have clearly shown that the natural compound DMDD possessed strong capability to prevent lung and liver tumor metastases, in addition to inhibiting primary breast tumor growth. Our findings suggest that DMDD inhibits tumor growth and metastasis by reducing the production of inflammatory cytokines, inducing apoptosis of the tumor cells, and blocking the TNF-α/NF-κB/MMPs pathways. These findings demonstrate the therapeutic potentials of DMDD as a safe and efficient anti-tumor and anti-metastatic agent to treat metastatic breast cancer.

## Materials and Methods

### Chemicals and Reagents

DMDD was isolated as described by Gao *et al*.^29^ and dissolved in 1XPBS. Tumor necrosis factor alpha (TNF-α), interleukin 6 (IL-6), IL-12, transforming growth factor beta (TGF-β), and vascular endothelial growth factor (VEGF) ELISA kits were purchased from Bioworld Technology Inc. (Minneapolis, MN, USA). Antibodies against Bax, Bcl-2, cleaved caspase-3, cleaved caspase-9, MMP-2, and MMP-9 were purchased from Cell Signaling Technology (Danvers, MA, USA). Antibodies against p-NFκB p65 and p-IκBα were purchased from Bioworld Technology Inc. Horseradish peroxidase enzyme (HRP)-labeled goat anti-rabbit monoclonal antibody and 3,3′-Diaminobenzidine (DAB) were purchased from Beijing Golden Bridge Biotechnology (Beijing, China).

### Acute toxicology test

All animal tests in this study were conducted in compliance with the guidelines for laboratory animal use and care and the protocols were approved by the Committee for the Care and Use of Experimental Animals at Guangxi Medical University. All BALB/c mice used in this study were obtained from the Animal Center of Guangxi Medical University.

Twenty BALB/c mice with an average weight of 20 ± 2 g were used for the acute toxicology test. After three-day adaption, the mice were randomly divided into two groups, the DMDD group and the control group (n = 10). For the DMDD group, the mice were treated with 0.2 mL of 500 mg/mL DMDD, which is equivalent to a 5,000 mg/kg dose, by intragastric administration. For the control group, the same volume of PBS was administered orally. The mortality, behavioral and neurological changes, and poisoning symptoms, were observed closely for two weeks and recorded. The food intake of each mouse was recorded daily, and the body weight (g) of each mouse was recorded before and after each week of compound administration.

After two weeks of oral administration, blood samples were drawn via the eyes. The blood samples were divided into two parts for hematological test and blood chemistry test, respectively. Hematological parameters, including red blood cell (RBC), hemoglobin (HB), mean cell hemoglobin (MCH), mean cell hemoglobin concentration (MCHC), mean corpuscular volume (MCV), platelet (PLT), white blood cell (WBC), neutrophil ratio (NEUTR), and lymphocyte ratio (LYM), were measured using the first part of the blood samples with a Sysmex KX-21NV Analyzer (Sysmex Co., Hyogo, Japan). The second part of the blood samples were centrifuged at 3500 rpm/min at 4°C for 10 min, and the supernatant was collected and measured with a 200FR NEO automatic biochemical analyzer (Toshiba, Japan) for the following parameters: alanine transaminase (ALT), aspartate transaminase (AST), alkaline phosphatase (ALP), total protein (TP), albumin (ALB), creatinine (CRE), blood urea nitrogen (BUN), total cholesterol (Tch), TG, and glucose (GLU).

At the end of the experiment, the mice were sacrificed and the following organs were collected: heart, liver, lung and kidney. The organs were fixed with 10% formaldehyde and stained with HE, and the pathological changes were examined under an optical microscope (Leica, DM6000, Wetzlar, Germany).

### Mice survival test

Twenty-five female BALB/c mice were randomly divided into the following five groups (n = 5): vehicle group, doxorubicin (Dox) group, DMDD high dosage group (100 mg/kg) (DMDD-H), DMDD medium dosage group (50 mg/kg) (DMDD-M) and DMDD low dosage group (25 mg/kg) (DMDD-L). After three-day adaptation, the mice were injected subcutaneously on the right mammary fat pad with 1 × 10^6^ 4T1 cells resuspended in 0.1 mL PBS. The mice were treated with three different doses of DMDD by intragastric administration every day for fourteen days. For the vehicle group, 0.1 mL PBS was used instead. For the Dox group, mice were treated with 0.5 mg/kg of Dox by tail intravenous injection every week for two weeks. All mice were kept under the same conditions, and the survival time (days) for each mouse was recorded. Survival curves were made using Kaplan-Meier plots, and the statistical difference was evaluated using the log-rank test.

### 4T1 Metastatic Mammary Carcinoma Model and *in vivo* Anti-tumor and Anti-metastasis Efficacy

Sixty female BALB/c mice of 5–6 weeks old were were randomly divided into the following six groups (n = 10): naïve group, vehicle group, doxorubicin (Dox) group, DMDD high dosage group (100 mg/kg) (DMDD-H), DMDD medium dosage group (50 mg/kg) (DMDD-M) and DMDD low dosage group (25 mg/kg) (DMDD-L). All mice except for those in the naïve group were injected subcutaneously on the right mammary fat pad with 10^6^ 4T1 cells resuspended in 0.1 mL PBS. After injection, their body weight and tumor size were measured every day. When tumor volumes reached about 300 mm^3^, the mice were treated with three different doses of DMDD by intragastric administration every day for fourteen days. For the vehicle group, 0.1 mL PBS was used instead. For the Dox group, mice were treated with 0.5 mg/kg of Dox by tail intravenous injection every week for two weeks.

After treatment, the mice were anesthetized and killed by cervical dislocation. The mammary tumors were carefully excised, and the maximum length (a) and minimum length (b) of the tumors were measured using an electronic digital caliper (accuracy: 0.01mm). Tumor volumes (V) were calculated using the formula: V = a × b^2^/2^3^.

The weight of the tumors were measured and the inhibition rates of tumor growth were calculated using the formula: Inhibition rate of tumor growth (%) = (Mean tumor weight of vehicle group − Mean tumor weight of treatment group)/Mean tumor weight of vehicle group × 100%.

The organs (lung, liver, and spleen) were harvested and processed immediately, and the serum was collected for cytokine analysis. The weight of the spleens were measured and the spleen indexes were calculated using the formula: Spleen index (%) = spleen weight / body weight × 100%. To assess metastasis, lung and liver tissues were used for histological and immunohistochemical analysis, and the metastatic nodules in lung tissues were counted.

Cell culture and cell viability assay. The 4T1 murine mammary carcinoma cell line was purchased from American Type Culture Collection (ATCC) (Manassas, VA, USA) and maintained in RPMI 1640 medium supplemented with 10% FBS and 1% penicillin-streptomycin. Cells were incubated in a humidified atmosphere with 5% CO_2_ at 37 °C. The cell viability assay and IC_50_ determination were performed in 4T1 cells using AlarmaBlue dye as described previously^29^. The 4T1 cells were treated with different concentrations of DMDD for 48 h and stained with AlarmaBlue for 3 h. The fluorescent intensity of each well was measured at Ex/Em of 555/590 nm on a SpectraMax M5 microplate reader (Molecular Devices).

### Detection of Cytokines in the Serum

Enzyme-linked immunosorbent assay (ELISA) kits were used to determine the levels of the cytokines (TNF-α, IL-6, IL-12, TGF-β and VEGF) in the serum according to the manufacture’s instructions. In brief, serum obtained from mice was incubated in an antibody coated 96-well plate at room temperature for 2 h. Following incubation, the plate was washed three times. Afterwards, the plate was incubated with the biotinylated antibody at room temperature for 2 h and then washed three times. A streptavidin–HRP was added to the plate and incubated for 30 min. The plate was washed again five times, and the substrate was added to the plate. Once a colored product was formed, the reaction was terminated by the addition of stop solution and the absorbance was measured at 450 nm using the SpectraMax Plus384 microplate reader (Molecular Devices, Sunnyvale, CA).

### Histological Analysis

Mammary tumors, lung and liver tissues were fixed in 10% buffered formalin at room temperature for 48 h. Subsequently, the samples were embedded in paraffin and sectioned longitudinally at 5 μm. The tumor sections were stained with hematoxylin & eosin (H&E) and examined using a Leica DM6000 microscope. Images were taken using an Olympus BX53 F microscope (Tokyo, Japan) and analyzed with the Leica Application Suite (LAS) digital image processing software (Leica).

### Immunohistochemistry (IHC) Analysis

After deparaffinization with xylene and rehydration with absolute alcohol, the tumor sections were stained with anti-mouse antibodies against Bax (1:400), Bcl-2 (1:1000), cleaved caspase-3 (1:800), cleaved caspase-9 (1:50), CD31 (1:400), MMP-2 (1:100), MMP-9 (1:100), p-NFκB p65 (1:100) or p-IκBα (1:100), using an IHC staining method that has been described previously^39^. Images were taken using a Leica DM6000 microscope and analyzed using LAS imaging processing software.

### Statistics analysis

Results were expressed as mean ± SD. Statistical analyses were conducted using GraphPad Prism software version 6.0 (GraphPad Software, La Jolla, CA, USA). ANOVA test was used for statistical analysis. P-value < 0.05 was considered to be statistically significant.

## Electronic supplementary material

Supplementary data
