# Supplementary material for: 2-Dodecyl-6-methoxycyclohexa-2,5-diene-1,4-dione inhibits the growth and metastasis of breast carcinoma in mice
Source: Sci Rep. 2017 Jul 27;7:6704. doi: 10.1038/s41598-017-07162-3 (PMC5532290; doi:10.1038/s41598-017-07162-3)
Supplement: Supplementary file 1 — Supplementary data [file 41598_2017_7162_MOESM1_ESM.pdf]

2-Dodecyl-6-methoxycyclohexa-2,5-diene-1,4-dione inhibits the growth and metastasis of breast carcinoma in mice

Chunxia Chen[1], Zhihuan Nong[1], Qiuqiao Xie[1], Junhui He[1], Wene Cai[1], Xiuneng Tang[1], Xiaoyu Chen[1], Renbin Huang[1]*, Ying Gao[2]*

[1]Department of Pharmacology, Guangxi Medical University, Nanning, Guangxi 530021, China
[2]Department of Biology and Tennessee Center for Botanical Medicine Research, Middle Tennessee State University, Murfreesboro, TN 37132, USA

*Co-corresponding authors:
Renbin Huang, Tel: 86 0771 533 9805, E-mail: huangrenbin518@163.com
Ying Gao, Tel: 1 615 898 5339, E-mail: ying.gao@mtsu.edu

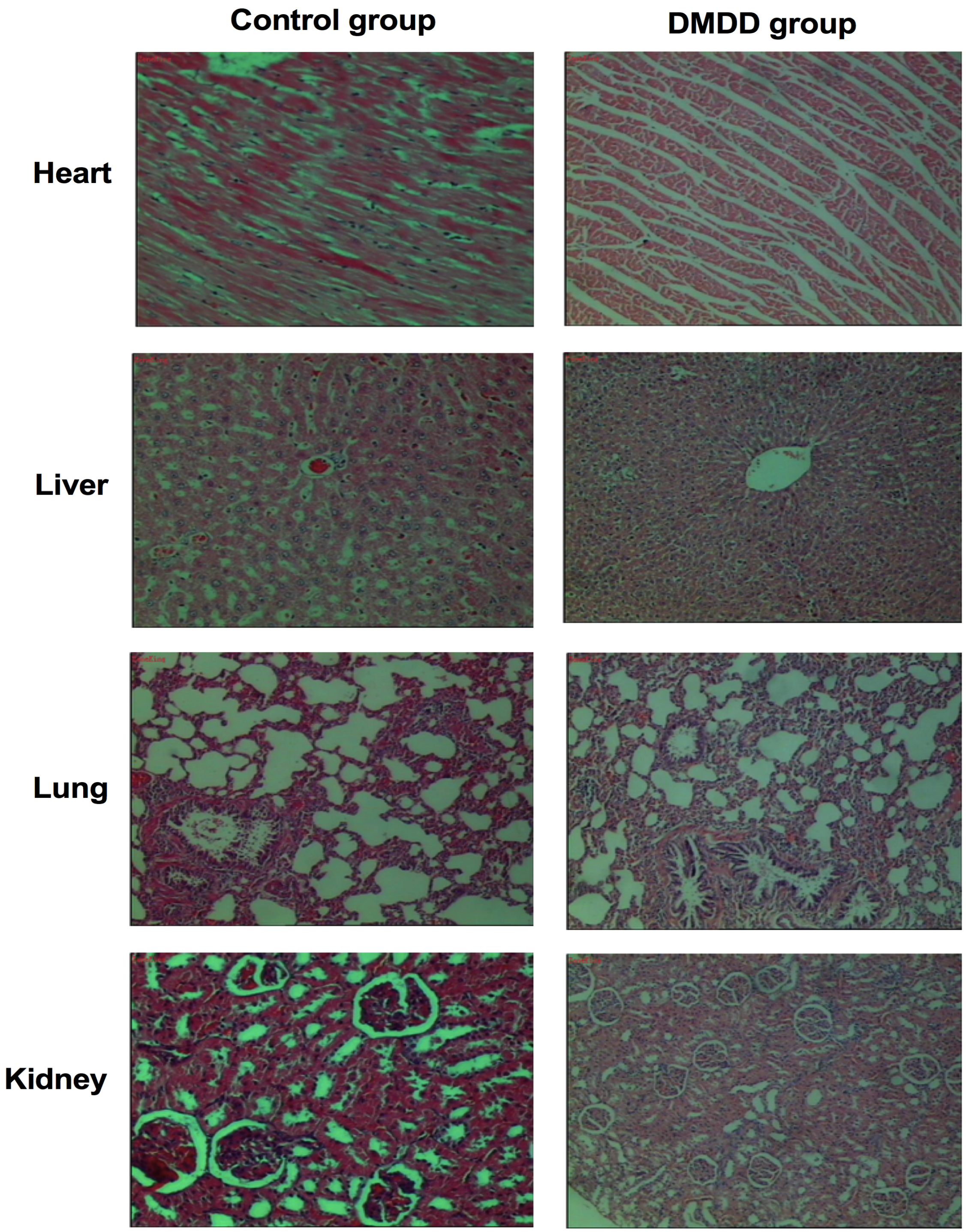

Supplementary Figure 1. Histopathological analysis of vital organs (heart, liver, lung and kidney) in mice. The tissues were dissected and stained with H&E, and observed under 400X microscope.
